# Changes in excitatory and inhibitory receptor expression and network activity during induction and establishment of epilepsy in the rat Reduced Intensity Status Epilepticus (RISE) model

**DOI:** 10.1016/j.neuropharm.2019.107728

**Published:** 2019-11-01

**Authors:** Hope I. Needs, Benjamin S. Henley, Damiana Cavallo, Sonam Gurung, Tamara Modebadze, Gavin Woodhall, Jeremy M. Henley

**Affiliations:** a School of Biochemistry, Centre for Synaptic Plasticity, Biomedical Sciences Building, University of Bristol, Bristol, BS8 1TD, UK; b School of Life and Health Sciences, Aston University, Aston Triangle, Birmingham, B4 7ET, UK

**Keywords:** Temporal lobe epilepsy, AMPA receptors, Synapse, Reduced intensity *status epilepticus* (RISE) model

## Abstract

The RISE model is an effective system to study the underlying molecular and cellular mechanisms involved in the initiation and maintenance of epilepsy *in vivo*. Here we profiled the expression of excitatory and inhibitory neurotransmitter receptor subunits and synaptic scaffolding proteins in the hippocampus and temporal lobe and compared these changes with alterations in network activity at specific timepoints during epileptogenesis. Significant changes occurred in all of the ionotropic glutamate receptor subunits tested during epilepsy induction and progression and the profile of these changes differed between the hippocampus and temporal lobe. Notably, AMPAR subunits were dramatically decreased during the latent phase of epilepsy induction, matched by a profound decrease in the network response to kainate application in the hippocampus. Moreover, decreases in the GABA_A_β3 subunit are consistent with a loss of inhibitory input contributing to the perturbation of excitatory/inhibitory balance and seizure generation. These data highlight the synaptic reorganisation that mediates the relative hypoexcitability prior to the manifestation of seizures and subsequent hyperexcitability when spontaneous seizures develop. These patterns of changes give new insight into the mechanisms underpinning epilepsy and provide a platform for future investigations targeting particular receptor subunits to reduce or prevent seizures.

## Highlights

• Changes in synaptic protein levels provide insight into synaptic reorganisation in epilepsy.
• In the RISE model changes in GluR subunits occur at different stages of epileptogenesis.
• Changes appear to occur independently of changes in synapse number.
• GluR profiles are directly linked to changes in network excitability.
• Targeting GluR subunits could provide a new strategy to reduce seizures.

## 1 Introduction

Epilepsy is characterised by recurrent seizures caused by excessive neuronal activity in susceptible brain regions. Up to ~1% of the population worldwide suffer from epilepsy, equating to ~50 million people and, of these, ~40% have Temporal Lobe Epilepsy (TLE). Although epilepsy can often be controlled with medication, ~30% of epilepsy cases are resistant to anti-epileptic drugs (AEDs) (WHO, 2018). Furthermore, approximately a third of those initially responsive to AEDs will transition to drug-resistant epilepsy (DRE) during the course of the disease (Devinsky et al., 2018).

There is an extensive, and sometimes contradictory, literature surrounding epileptogenesis and progression of TLE (for recent reviews see (Becker, 2018; Fukata and Fukata, 2017)). Notwithstanding the many unresolved questions, it is now widely accepted that synaptic function is dynamically scaled in response to activity (homeostatic synaptic scaling) and that dysfunction of receptors and/or their signalling pathways play key roles in disrupting the excitatory/inhibitory balance that underpins epilepsy (Badawy et al., 2012).

Systemic or local insult can cause vulnerable brain regions to develop spontaneous recurrent seizures. Many studies assess changes during the *status epilepticus* period of intense seizure activity, which is often associated with high mortality and/or global damage to large areas of the brain. Reduced Intensity *Status Epilepticus* (RISE) is a low mortality, high morbidity rat model of chronic TLE characterised by a relatively long seizure-free (latent) period between induction and the development of spontaneous recurrent seizures (SRS) (Modebadze et al., 2016). Importantly, RISE replicates some of the core features of human temporal lobe epilepsy including restriction to temporal lobe structures, variation in seizure frequency and intensity between animals and slow periodic variations in seizure activity. Moreover, RISE avoids the gross neuronal damage that may be seen with alternative models, showing comparatively low levels of neuronal damage in the hippocampus (Modebadze et al., 2016).

There have, however, been no biochemical analyses of the expression levels of key neuronal proteins in RISE rats. To gain insight into molecular changes that occur during the initiation, development and establishment of epilepsy, we systematically profiled an array of synaptic receptor proteins in the hippocampus and the temporal lobe of RISE rats and non-epileptic age-matched controls (AMC). The time points sampled were: 24 h after injection with pilocarpine, when rats are recovering from the initial *Status Epilepticus* (***SE***) phase; 2–4 weeks after pilocarpine injection, when the rats are in a latent period (***LP***); and the chronic phase following the onset of spontaneous recurrent seizures (***SRS***), usually 3+ months after pilocarpine injection.

There were changes in many of the proteins tested, most of which display reduced levels in RISE compared to AMC rats. In particular, we detected highly significant reductions in levels of AMPAR subunits. However, the expression levels of some proteins, notably the NMDAR subunit GluN1 and the excitatory synaptic marker PSD95, increased at specific phases of epilepsy. The alterations in protein levels throughout epilepsy progression differed between the hippocampus and the temporal lobe. Moreover, the changes in receptor expression were consistent with alterations in neuronal network excitability in hippocampal area CA3, as measured using a kainate challenge protocol that defines peak excitability in response to application of 100 nM kainate against a baseline measure of network oscillatory power made without kainate application.

## 2 Materials and methods

### 2.1 Generation of the *In Vivo* RISE model of epilepsy

RISE rats were generated at Aston University as reported previously (Modebadze et al., 2016). Detailed methods are provided in Supplemental Material. Three timepoints during epilepsy progression were sampled in RISE rats and age-matched controls (AMC):

1) *Status Epilepticus* (***SE***); rats were sacrificed 24 h post-induction with pilocarpine.
2) Latent period (***LP***); rats were sacrificed 2–4 weeks after SE induction, corresponding to the latent, seizure-free phase of epilepsy.
3) Spontaneous recurrent seizures (***SRS***); rats were sacrificed 3+ months post-induction of SE immediately following the development of spontaneous recurrent seizures, as determined by behavioural tests and/or video recording of behaviour (Modebadze et al., 2016).

### 2.2 Western blotting

Samples of hippocampus and temporal lobe dissected from four or five individual RISE and AMC rats containing 40 μg of total protein were run in parallel on the same 10% SDS-PAGE gel and immunoblotted along with loading controls of either β-actin, β-tubulin III or α-GAPDH, depending on the MW of the protein investigated. At least two technical repeats were performed. After protein transfer, PVDF membranes were blocked and probed with appropriate primary and secondary antibody (Supplemental Material, Table 1) and quantitatively imaged using an Odyssey Imaging System (LI-COR). Importantly, this dymanic quantitification system removes the possibility of any error due to overexposure of the Western blot that can occur using film based analysis.

### 2.3 Electrophysiology

Animals were taken at specific timepoints during epileptogenesis and brain slices prepared using standard techniques. Spontaneous gamma (SγO) and kainic acid-induced (KγO) oscillations were recorded from CA3 of the hippocampus. Detailed methods are provided in the Supplemental Material. Animals were visusally monitored prior to, and during, anaesthesia to ensure that acute seizures did not occur within the 60 minutes immediately before preparation for slices or blotting.

### 2.4 Statistical methods

Standard statistical tests were used and further details are provided in the Supplemental Material.

## 3 Results

### 3.1 Synaptic marker proteins

**Fig. 1:**
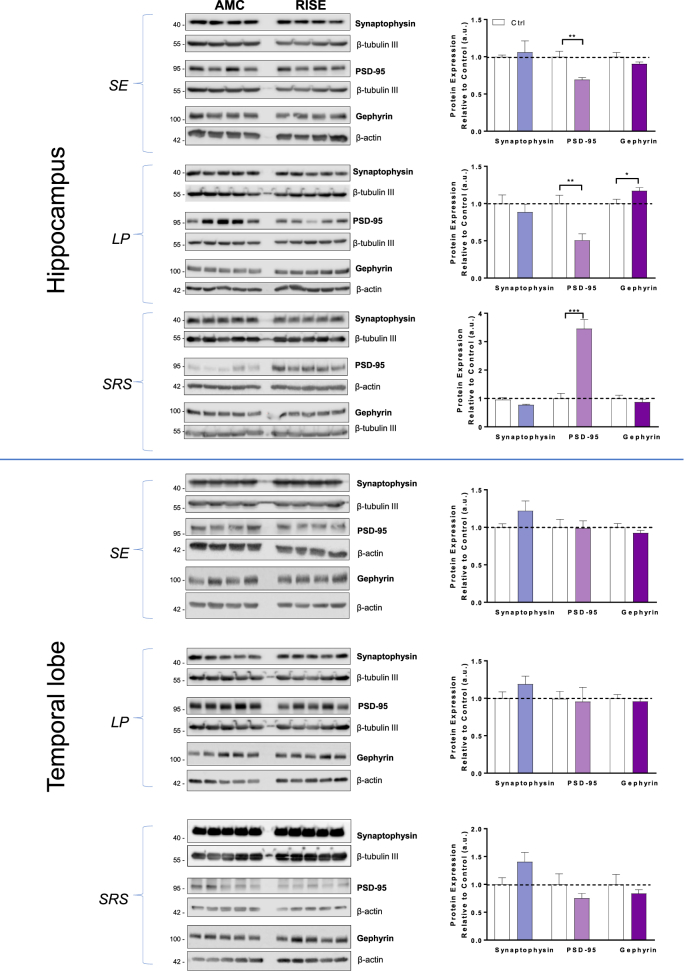
**Levels of synaptic marker proteins in hippocampus and temporal lobe in AMC and RISE rats at different stages of epileptogenesis.** RISE rats were sacrificed 24 h (*SE*), 2–4 weeks (*LP*) or ~3+ months (*SRS*) post pilocarpine injection and hippocampi and temporal lobes dissected, immediately flash frozen in liquid nitrogen and stored at −80 °C until use. Samples were homogenised, protein matched and 40 μg of total protein was loaded per lane. Left panels: Representative Western blots of hippocampal and temporal lobe samples from 4 (*SE*) or 5 (*LP* and *SRS*) separate AMC and RISE rats immunoblotted for synaptophysin, PSD95 and gephyrin. β-tubulin III and β-actin were used as loading controls. Right panels: Quantification of immunoreactive bands normalised to their respective loading controls. Error bars represent the mean ± SD from 4 samples, with the mean of the control group set to 1. *P < 0.05 vs Ctrl, **P < 0.01 vs Ctrl, ***P < 0.001 vs Ctrl (Unpaired *t*-test).

We first investigated levels of the presynaptic marker synaptophysin, the excitatory postsynaptic marker PSD95 and the inhibitory postsynaptic marker gephyrin in hippocampus and temporal lobe samples at *SE*, *LP* and *SRS* stages of epilepsy (Fig. 1). There were no significant changes in synaptophysin in the hippocampus or temporal lobe at any stage of epilepsy. Similarly, there were no differences in PSD95 or gephyrin levels at any timepoint in the temporal lobe. However, there were highly significant changes in the levels of PSD95 in the hippocampus where PSD95 decreased by ~30% in *SE* and ~50% in *LP* compared to AMC but, in stark contrast, there was a 3-fold increase in *SRS* (Fig. 1). Interestingly, we also detected a significant increase in gephyrin during the latent phase (*LP*) but no change in any other stage in the hippocampus.

### 3.2 Neurotransmitter receptors

**Fig. 2:**
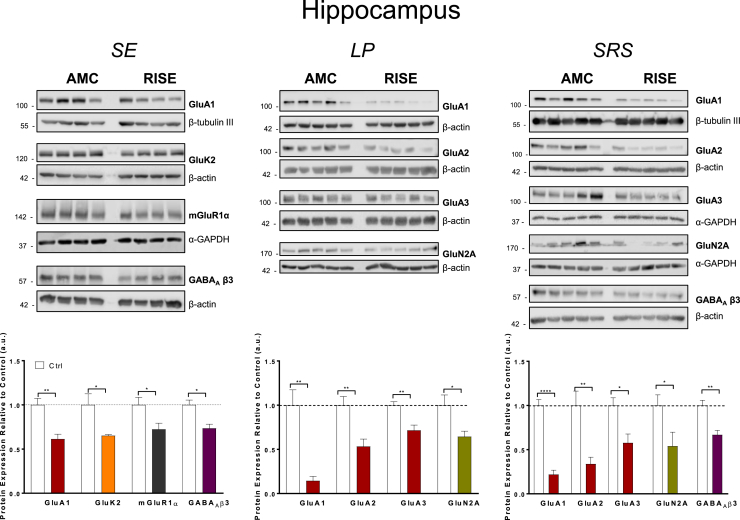
**Levels of receptor proteins in hippocampus that showed significant changes between AMC and RISE rats at different stages of epileptogenesis.** Top panels: Representative Western blots of hippocampal samples immunoblotted for the AMPAR subunits GluA1, GluA2 and GluA3, KAR subunit GluK2, NMDAR subunit GluN2A, Group 1 metabotropic glutamate receptor subtype mGluR1α, and the GABA_A_R subunit β3. β-tubulin III, β-actin and α-GAPDH were used as loading controls. Bottom panels: Quantification of immunoreactive bands normalised to their respective loading controls. Bars represent the mean ± SD from 4 to 5 samples, with the mean of the control group set to 1. *P < 0.05, **P < 0.01, ****P < 0.0001 vs Ctrl (Unpaired *t*-test).

**Fig. 3:**
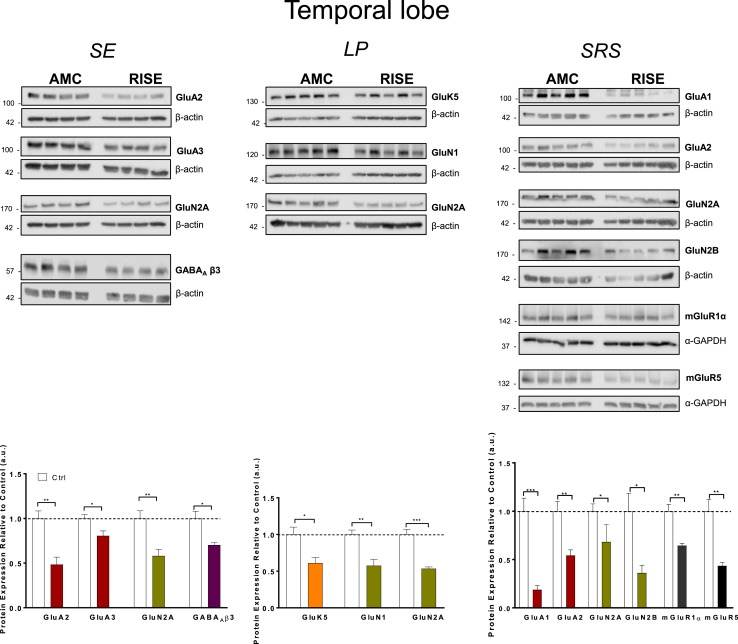
**Levels of receptor proteins in temporal lobe that showed significant changes between AMC and RISE rats at different stages of epileptogenesis.** Top panels: Representative Western Blots of hippocampal samples blotted for AMPAR subunits (GluA1, GluA2 and GluA3), KAR subunit GluK5, NMDAR subunits (GluN1, GluN2A, GluN2B), Group 1 metabotropic glutamate receptor subtypes (mGluR1α and mGluR5), and GABA_A_R subunit β3 (GABA_A_ β3). β-actin and α-GAPDH were used as loading controls. Bottom panels: Quantification of immunoreactive bands normalised to their respective loading controls. Error bars represent the mean ± SD from 4 to 5 samples, with the mean of the control group set to 1. *P < 0.05, **P < 0.01, ***P < 0.001 vs Ctrl (Unpaired *t*-test).

In parallel, we determined the levels of a range of neurotransmitter receptor proteins that have been implicated in epilepsy in hippocampus and temporal lobe samples. We measured total levels of the most abundant AMPAR and kainate receptor (KAR) subunits (GluA1, GluA2, GluA3 (Henley and Wilkinson, 2016) and GluK2, GluK5 (Evans et al., 2017) respectively); the obligatory NMDAR subunit GluN1 and the GluN2A and GluN2B subunits that play a role in determining NMDAR localisation and function (Paoletti et al., 2013); metabotropic glutamate receptors mGluR1α and mGluR5 (Suh et al., 2018); the presynaptic cannabinoid receptor CB1 (Busquets Garcia et al., 2016); and the most prevalent subunit of inhibitory GABA_A_Rs, GABA_A_β3 (Sieghart and Savic, 2018). Representative Western blots and quantification for proteins that showed changes in hippocampus and temporal lobe are presented in Fig. 2 and Fig. 3 respectively. Blots and quantification for proteins that did not change significantly are shown in Supplemental Figs. 1 and 2.

### 3.3 Hippocampus

#### 3.3.1 Timepoint 1; SE

Consistent with previously reported work on hippocampal sclerosis in epilepsy (Sendrowski and Sobaniec, 2013), we observed multiple changes in protein levels. Immediately following SE, the AMPAR subunit GluA1 was decreased by 40% but there were no corresponding reductions in GluA2 or GluA3 compared to AMC at this initial timepoint. These data suggest a selective loss of GluA1 containing AMPARs during early phase TLE, with minimal effects of GluA2/GluA3 heteromeric AMPARs.

There was also a ~35% reduction in the KAR subunit GluK2 with no significant change in GluK5. Levels of mGluR1α and GABA_A_β3 were also significantly reduced whereas no significant changes were detected for the NMDAR subunits GluN1, GluN2A or GluN2B (Supplemental Fig. 1).

#### 3.3.2 Timepoint 2; LP

During the latent phase the AMPAR subunits (GluA1, GluA2 and GluA3) were all dramatically reduced in the hippocampus. Remarkably, GluA1 was reduced by ~80% compared to AMC rats. In addition, although unchanged immediately following SE, levels of GluA2 and GluA3 reduced by ~50% and ~40% respectively during *LP*. Interestingly, after the initial *SE* timepoint, levels of GluK2 did not change compared to AMC rats, suggesting a recovery in expression of this KAR subunit. The only other significant change was a decrease in GluN2A, which was not observed in *SE*.

#### 3.3.3 Timepoint 3; SRS

At this timepoint, most RISE animals show electrographic and behavioural evidence of seizure activity and so it was surprising that levels of GluA1, GluA2 and GluA3 AMPAR subunits in the hippocampus remained very substantially reduced compared to AMC rats following the onset of spontaneous seizures. In addition, levels of GluN2A also remained decreased during *SRS* (Fig. 2 & Supplemental Fig. 1), while the levels of GABA_A_β_3_, that initially decreased in the SE phase but remained unchanged during LP phase, decreased significantly in this phase.

### 3.4 Temporal lobe

#### 3.4.1 Timepoint 1; SE

In contrast to our observations in the hippocampus, there was no significant decrease in the AMPAR subunit GluA1 but there were significant reductions in GluA2 and GluA3 (Fig. 3 & Supplemental Fig. 2). The only other significant changes at this timepoint in the temporal lobe were decreases in GluN2A and GABA_A_β3.

#### 3.4.2 Timepoint 2; LP

During the latent period none of the AMPAR subunits were altered compared to AMC samples. However, GluN1 decreased by ~50% in RISE compared to AMC rats along with a significant decrease in GluN2A. Since GluN1 is obligatory for functional NMDAR complexes we speculate that this would lead to reduced NMDAR-mediated synaptic transmission during *LP*.

In addition, we detected a 40% decrease in levels of the KAR subunit GluK5 but no change in GluK2, consistent with a change in the subunit stoichiometry of KAR complexes in the temporal lobe during the latent period.

#### 3.4.3 Timepoint 3; SRS

Despite being unchanged during *LP*, temporal lobe levels of two of the AMPAR subunits (GluA1 and GluA2, but not GluA3) were substantially decreased in *SRS*. Indeed, similar to the hippocampal samples, GluA1 was reduced by ~80% compared to AMC rat levels. Correspondingly, GluA2 was also significantly reduced. In addition, mGluR1α and mGluR5 were significantly decreased, as were levels of the NMDAR subunits GluN2A and GluN2B, but not of GluN1 (Fig. 3).

### 3.5 Overview of changes detected in the three stages of epilepsy in RISE rats

**Fig. 4:**
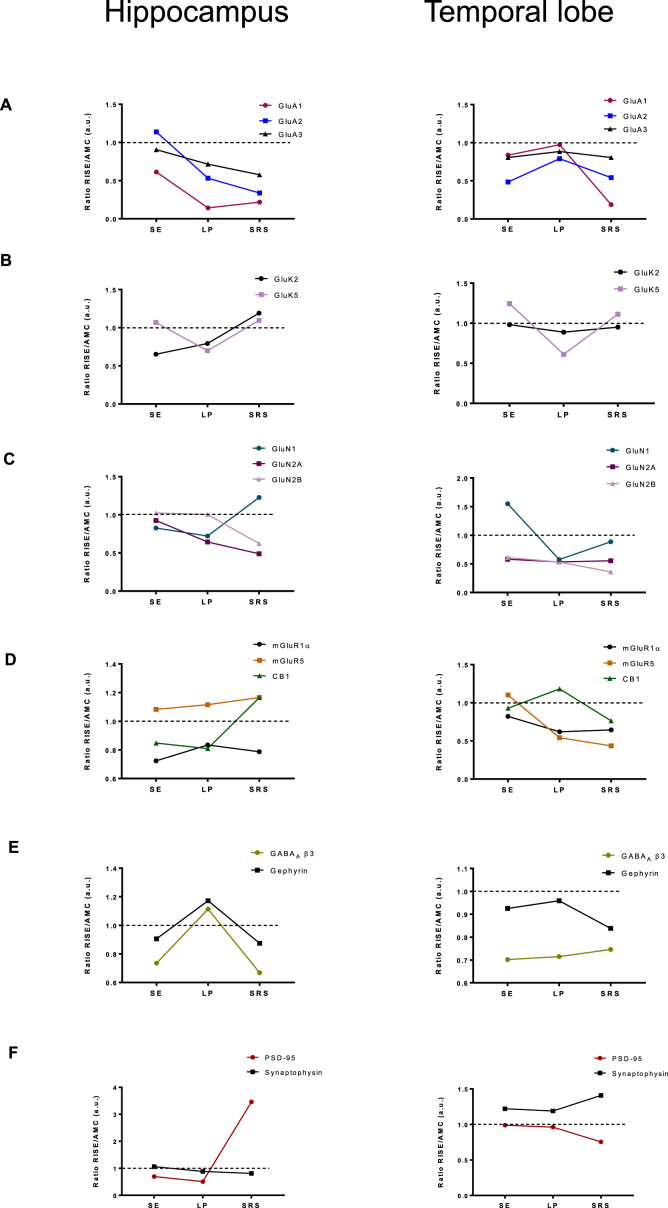
**Profile of expression level changes for each protein tested at different stages of epileptogenesis.** Ratio between average RISE and AMC immunoblot intensity values in both the hippocampus and the temporal lobe. For each set of samples (*SE*, *LP*, *SRS*) the mean value of the AMC immunoblots was set to 1 (dotted line) and the mean of the corresponding RISE data plotted to illustrate the profile of change at different stages of epileptogenesis in the hippocampus and the temporal lobe. **(A)** Ratio of GluA1, GluA2 and GluA3 subunit levels. **(B)** Ratio of GluK2 and GluK5 subunit levels. **(C)** Ratio of GluN1, GluN2A and GluN2B subunit levels. **(D)** Ratio of mGluR1α, mGluR5 and CB1 receptor levels. **(E)** Ratio of GABA_A_ β3 subunit and gephyrin (inhibitory synapse marker) levels. **(F)** Ratio of synaptophysin and PSD95 (excitatory synapse markers) levels.

An overview of the profiles of changes in RISE compared to AMC rats is shown in Fig. 4. However, prominent exceptions were the pronounced increase in PSD95 in the hippocampus, but not in the temporal lobe, in *SRS* as well as the timing of the differential loss in AMPAR subunits between these two brain regions.

Our data highlight the fact that GABA_A_β3 and gephyrin have a very similar expression profile in the hippocampus. More specifically, these inhibitory synapse proteins are decreased during active seizure stages (*SE* and *SRS*) but not during the seizure free *LP* stage of epilepsy. Taken together, these data are consistent with a deficit in inhibitory neurotransmission contributing to seizure activity.

### 3.6 Correlation between changes in receptor and synaptic marker protein abundance

**Fig. 5:**
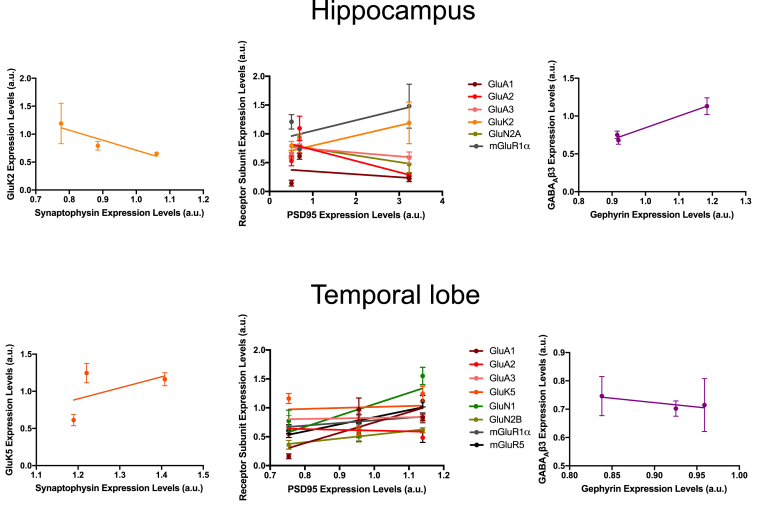
**Correlation between changes in receptors and synaptic marker proteins in hippocampus and temporal lobe at different stages of epileptogenesis.** **(A)** Scatterplot showing no correlation between levels of the pre-synaptic protein synaptophysin and GluK2 (KARs have both pre- and postsynaptic localisation) in hippocampus (r = −0.921, p = 0.2547, n = 5). **(B)** Scatterplot showing no correlation between levels of the post-synaptic protein of excitatory synapses PSD95 and significantly altered post-synaptic receptor subunits in hippocampus. GluA1 (r = −0.309, p = 0.8000, n = 5), GluA2 (r = −0.7034, p = 0.5033, n = 5), GluA3 (r = −0.5376, p = 0.6387, n = 5), GluK2 (r = 0.9502, p = 0.2017, n = 5), GluN2A (r = −0.751, p = 0.4591, n = 5), and mGluR1⍺ (r = 0.736, p = 0.4735, n = 5). **(C)** Scatterplot showing no correlation between gephyrin, a post-synaptic scaffold at inhibitory synapses, and levels of GABA_A_β3 in hippocampus (r = 0.9875, p = 0.1007, n = 5). **(D)** Scatterplot showing no correlation between levels of pre-synaptic protein synaptophysin and the KAR subunit GluK5 in temporal lobe (r = 0.5097, p = 0.6595, n = 5). **(E)** Scatterplot showing no correlation between levels of PSD95 and significantly altered post-synaptic receptor subunits in temporal lobe. GluA1 (r = 0.7936, p = 0.4164, n = 5), GluA2 (r = −0.1451, p = 0.9073, n = 5), GluA3 (r = 0.3037, p = 0.8036, n = 5), GluK5 (r = 0.09309, p = 0.9406, n = 5), GluN1 (r = 0.7315, p = 0.4777, n = 5), GluN2A (r = −0.7079, p = 0.4993, n = 5), GluN2B (r = 0.9824, p = 0.1198, n = 5), mGluR1⍺ (r = 0.9415, p = 0.2189, n = 5), and mGluR5 (r = 0.8046, p = 0.4047, n = 5). **(F)** Scatterplot showing no correlation between levels of gephyrin and GABA_A_β3 in temporal lobe (r = −0.8509, p = 0.3521, n = 5).

To exclude the possibility that the differences in receptor proteins simply reflected changes in the numbers of synapses, we performed correlation analyses. As shown by the line plots in Fig. 5 (statistical analysis in Supplemental Table 2), there was no correlation between changes in synaptic marker proteins and receptor proteins. It should be noted that Western blotting captures total levels of protein in the cell and that only a proportion of neurotransmitter receptors are surface expressed at any particular time; for example ~50% for GluA2 (Perestenko and Henley, 2006). Moreover, because surface expressed receptors can be highly laterally mobile within the plasma membrane, at any given timepoint and depending on the synaptic activity, only a sub-population of AMPARs are present at synapses. Thus, it is not surprising that our data imply that changes in total levels of receptor proteins occur independently of changes in synapse number. These results are consistent with the changes in receptors we observe playing mechanistic causal roles in, rather than simply being a downstream consequence of, the epileptogenic process and neuronal feedback responses to increased activity.

### 3.7 GluR-mediated network excitability in hippocampal area CA3

**Fig. 6:**
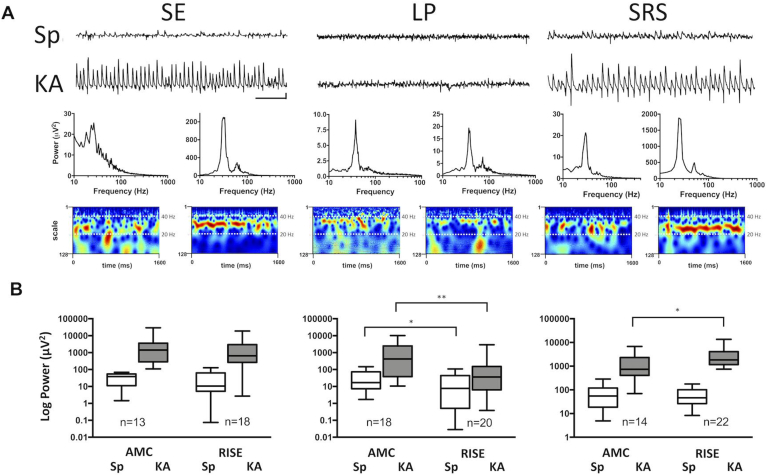
**Electrophysiological analyses of network activity during epileptogenesis.** **(A)** Representative raw data traces of spontaneous (Sp) and KA-evoked (KA) gamma activity from each timepoint under study with associated power spectra and Morlet-wavelet spectrograms in slices from epileptic groups. Scale bars for representative raw traces 200 ms and 100 μV (right and left panels), 200 ms and 50 μV (middle panel). **(B)** Summary box and whisker plots of pooled data from groups in each epileptic condition and from control groups. *P < 0.05, **P < 0.01 (Mann-Whitney test).

To explore the functional implications of the observed changes in receptor subunits, we monitored the excitability of CA3 networks in 105 horizontal hippocampal-entorhinal slices taken from 86 animals. We compared both the baseline neuronal network oscillatory activity (spontaneous gamma oscillations; SγO) in AMC vs. epileptic slices and similar network activity following 100 nM kA application (kainate gamma oscillations; KγO) in the same slices. The ratio of the power of KγO:SγO was used as an indicator of the amount of AMPAR/KAR excitation that could be produced in a given slice. Representative raw traces and power spectra of both oscillation types are shown in Fig. 6A. In slices from RISE rats at timepoint 1 (24h, *SE*), the SγO exhibited concurrent high-frequency bursts and γ rhythm activity. In contrast, oscillations in AMC slices (not shown) were more stable and synchronized, remaining within a relatively narrow frequency band.

More specifically, analysis using the Morlet-wavelet spectrogram revealed a mix of slow and fast oscillations in the RISE slices (Fig. 6A, leftmost panel). Although SγO seemed abnormal in RISE rats just after *SE*, the network was still responsive to 100 nM kA stimulation, which caused a large increase in power and produced more coherent synchronised oscillations (Fig. 6A, leftmost panel), comparable to those observed in AMC slices in the presence of KA (see pooled data in Fig. 6B). Interestingly, KA suppressed high-frequency activity in epileptic slices.

Pooled data demonstrate that SγO mean peak power in the RISE *SE* slices was comparable to AMC slices (36.6 ± 10.1 μV^2^, n = 18, vs. 33.7 ± 6.8 μV^2^,n = 13, ns) while the mean peak frequency was higher than in the AMC slices (30.5 ± 1.1 Hz, n = 18, vs. 26.7 ± 0.8 Hz, n = 13, p < 0.05). In the presence of 100 nM kA, RISE *SE* slices produced KγO similar to AMCs in mean peak power (3235 ± 1333 μV^2^, n = 18, vs. 4024 ± 2191 μV^2^, n = 13, p > 0.05), although higher in mean peak frequency (36.2 ± 1.4 Hz, n = 18, vs. 29.5 ± 1.4 Hz, n = 13, p < 0.01) (Fig. 6B).

Our Western blots indicate that AMPAR subunits are profoundly decreased in the hippocampus during the latent period. We therefore specifically compared the SγO and KγO in RISE and AMC slices at this timepoint. Under basal conditions in RISE slices the SγO was weak and inconsistent over time, indicating underlying abnormalities of the network function (Fig. 6A, middle panel). Moreover, spontaneous activity was low in power but coherent gamma was observed, with a sharp peak on the power spectrum, not normally characteristic of low-power SγO. AMC slices, on the other hand, consistently exhibited strong SγO in CA3, indicative of normal hippocampal neuronal network function (Fig. 6A, middle panel).

When we challenged slices in the *LP* timepoint with KA, profound differences in network excitability were apparent. In AMC slices, 100 nM kA elicited KγO with a dramatic increase in the power compared to SγO, whereas in RISE slices KA did not significantly change the network activity. Overall, RISE slices showed significantly lower SγO power compared to AMC slices at this timepoint (20.6 ± 6.6 μV^2^, n = 20, vs. 41 ± 11.5 μV^2^, n = 18 p < 0.05), although oscillations were similar in mean peak frequency (34.9 ± 1.2 Hz, n = 20, vs. 35.4 ± 0.9 Hz, n = 18 p > 0.05). Furthermore, KγO mean peak power in RISE slices was considerably lower than in AMC slices during *LP* (322.5 ± 156.7 μV^2^, n = 20, vs. 1968 ± 756.8 μV^2^, n = 18 p < 0.01), although no significant difference was observed in mean peak frequency (36.4 ± 1.3 Hz, n = 20, vs. 34.6 ± 0.8 Hz, n = 18 p > 0.05). Taken together these data support the contention that decreased AMPAR and KAR expression during the *LP* is associated with a profound decrease in network excitability.

To determine whether rhythmic activity in the hippocampus underwent further changes during epileptogenesis, we explored SγO and KγO in slices from chronically epileptic rats (*SRS*) and compared them to AMC. SγO demonstrated strong power in both control and epileptic slices, although concurrently with gamma frequency *SRS* slices exhibited transient high-frequency activity. When 100 nM KA was applied to pre-existing SγO, rhythmic activity dramatically increased in power and became regular in both groups (Fig. 6, rightmost panel). Moreover, in epileptic slices administration of KA abolished fast activity and introduced a pure gamma frequency rhythm. The main parameters of SγO were comparable in *SRS* vs. AMC slices (62.7 ± 9.6 μV^2^ at 35.9 ± 0.9 Hz, n = 22, vs. 84.3 ± 23.9 μV^2^ at 36.3 ± 0.9 Hz, n = 14, p > 0.05), as shown in Fig. 6B. KγO, on the other hand, were significantly stronger in power in *SRS* slices compared to AMC (3401 ± 757.4 μV^2^, n = 22, vs. 1798 ± 612.7 μV^2^, n = 14, p < 0.05). There was no significant difference in the frequency of KγO between epileptic and AMC slices (29.9 ± 0.8 Hz, n = 22, in RISE vs. 31.4 ± 0.4 Hz, n = 14, in AMC, p > 0.05). These results suggest elevated excitability of CA3, which may be linked to the appearance of behavioural seizures during this period.

## 4 Discussion

Dysregulation of the inhibitory/excitatory balance leading to excessive excitatory synaptic transmission, synaptic reorganisation and aberrant changes in neuronal circuits all contribute to the initiation and onset of epilepsy (Bozzi et al., 2012). However, the profiles of how key proteins change, which are important for insight into the mechanisms of synaptic reorganisation and network reorganisation that lead to TLE onset and maintenance, are not fully characterised. In this study, we investigated how levels of specific proteins implicated in TLE change in RISE rats during onset (*SE*), latent (*LP*) and chronic (*SRS*) phases of epilepsy.

### 4.1 Synaptic marker proteins

Our data highlight alterations in the levels of synaptic markers in both the hippocampus and temporal lobe during epileptogenesis. These changes vary between the brain regions tested as well as the stage of epilepsy, consistent with profound and dynamic changes in neuronal network connectivity.

There were dramatic changes in PSD95 in the hippocampus during epileptogenesis, not observed in the temporal lobe. At *SE* and *LP* stages PSD95 was decreased but during *SRS* there was a 3-fold increase in PSD95 compared to AMCs. The increase in PSD95 in chronic epilepsy may be due to a reactive process driving the formation of more postsynaptic components at each given presynaptic terminal. We considered that these changes might represent compensation for the loss of AMPARs by providing more synaptic scaffolding for their recruitment, but further analysis (Fig. 5) did not reveal a correlation between total levels of AMPAR subunits and total levels of PSD95. The fact that we did not see changes in synaptophysin is perhaps surprising since it is well established that glutamatergic neurons in the hippocampus undergo axonal sprouting during *SRS*. However, we analysed whole hippocampi and sprouting does not occur across all subregions, being confined mainly to dentate gyrus and some cells within CA1 (Esclapez et al., 1999; Nadler et al., 1980).

In contrast to the decrease seen for PSD95, there was an increase in gephyrin during the *LP*. Overall, we saw a rebound increase in excitatory synapse marker during *SRS* with no change in inhibitory synapse markers, consistent with electrophysiologically determined changes in the excitation/inhibitory balance.

### 4.2 Glutamate receptors

The temporal profiles of changes in AMPAR subunits between hippocampal and temporal lobe samples differed markedly. In the hippocampus GluA1 was decreased in all three phases of epilepsy, with only very low levels present in *LP* and *SRS*. In contrast, in the temporal lobe, GluA1 was only significantly decreased during the *SRS* phase. This broadly agrees with a previous report of GluA1 downregulation in TLE (Fukata and Fukata, 2017), and is consistent with dysfunctional regulation of GluA1 being an important factor in both epileptogenesis and the spread of seizures across the brain.

In the hippocampus, GluA2 and GluA3 subunits were not decreased immediately following *SE* but were both significantly reduced during *LP* and *SRS*. In the temporal lobe GluA2 and GluA3 were reduced after *SE*, though returned to AMC levels during *LP*. GluA1 and GluA2 subunits were decreased during *SRS*, but GluA3 was not. Consistent with these changes it has been reported recently that there are significant reductions in GluA1 and GluA2 in synaptosomal fractions from a KA-based rat model of TLE (Egbenya et al., 2018). The complex patterns of changes to individual subunits suggests dynamic rearrangement of AMPAR composition and properties at different phases of epileptogenesis.

It should be noted, however, that a recent study has reported that there are regional differences in AMPARs within the hippocampus (Joshi et al., 2017). More specifically, in animals in self-sustaining limbic SE the amplitudes of the miniature, spontaneous, and AMPA-evoked excitatory currents recorded from the CA1 pyramidal neurons were enhanced. The surface expression of GluA1 subunit-containing AMPARs, assessed by biotinylation of hippocampal slices and subsequent microdissection, was increased in the CA1 pyramidal neurons whereas it was unchanged in dentate granule cells (DGCs) (Joshi et al., 2017). Thus, it is possible that our use of the whole hippocampus for total AMPAR subunit levels may overlook distinct changes in sub-regions of the hippocampus. Future, more refined experiments are therefore needed to assess how different AMPAR subunits are surface expressed in the RISE model.

Kainate has long been used in epilepsy research to induce seizures (Falcon-Moya et al., 2018). However, the only condition in which total levels of the KAR subunit GluK2 were decreased was immediately following *SE* in the hippocampus. Levels of GluK5 were only significantly decreased in the latent stage (*LP*) of epilepsy in the temporal lobe but selective GluK5 antagonists have been reported to prevent convulsive activity and seizures in pilocarpine-induced epilepsy (Smolders et al., 2002). Thus, given the strong links between KARs and epilepsy, we speculate that it is likely that changes in the number and/or stoichiometry of KARs at the cell surface, rather than the total amount of KAR subunit protein, may be a determining factor in epileptogenesis.

We did not detect significant changes in any of the NMDAR subunits immediately following *SE*, however, GluN2A was decreased in both the hippocampus and temporal lobe during *LP*. GluN1 expression was also decreased at this stage in the temporal lobe. Intriguingly, GluN2A remained decreased in the hippocampus during *SRS*, while in the temporal lobe both GluN2A and GluN2B were decreased. Different NMDAR populations can have synaptic/extrasynaptic distributions and respond differently to activity, which may provide a partial explanation for the differential expression of NMDAR subunits highlighted in our analyses (Sanz-Clemente et al., 2013). Overall our data from RISE suggest a decrease in NMDAR mediated excitatory drive.

### 4.3 GABA_A_Rs

The GABA_A_β3 subunit was decreased immediately following *SE* and again in *SRS* in the hippocampus, and immediately following *SE* in the temporal lobe. This is consistent with a loss of inhibitory input contributing to the perturbation of excitatory/inhibitory balance and seizure generation in *SE*. Many antiepileptic drugs function through GABAergic mechanisms and evidence for altered GABA_A_R function and/or subunit expression in epilepsy is well established. Consistent with this, one interpretation of our data is that the changes we observed could correspond to reported GABA_A_R internalization/degradation during seizures (Goodkin et al., 2007; Kapur and Macdonald, 1997) that may be related to the recognized loss of GABAergic interneurons which underlies changes in excitability during epileptogenesis.

### 4.4 Network excitability

Our data strongly support the notion that changes in receptor expression are directly linked to alterations in network excitability. It is tempting to speculate that loss of GluA1 in the latent period is at least partly responsible for relative hypoexcitability in CA3 during this time. Similarly, the increase in PSD95 may reflect a synaptic mechanism for hyperexcitability at *SRS*, despite the lower expression of AMPAR subunits.

However, it is also possible that AMPAR hypofunction due to the profound reduction in GluA1, GluA2 and GluA3 expression is directly responsible for epileptiform activity. For example, in *anti*-AMPAR autoimmune encephalitis, antibodies generated against AMPAR epitopes lead to AMPAR internalization and temporal lobe seizures in humans (Gleichman et al., 2014; Haselmann et al., 2018; Lai et al., 2009). Similarly, *anti*-NMDAR autoantibodies cause both receptor internalisation and seizures (for review see (Dalmau et al., 2017)). Clearly, the balance between excitation and inhibition in neuronal networks involves subtleties that preclude simplistic classification of receptor changes as pro- or anti-convulsant, for example, loss of excitatory drive to GABA interneurons certainly has the potential to be an important mechanism for seizure generation (Chun et al., 2019). Thus, even for AMPARs the role(s) of different subunits and multiple post-translational modifications in LTP, LTD and homeostatic synaptic up- and down-scaling are only just being classified and understood in terms of ‘the AMPAR code’ (Diering and Huganir, 2018). Further work on the role of AMPAR dynamics in epileptogenesis will require detailed analyses at both cell- and synapse-specific levels.

Finally, the loss of hippocampal GABA_A_β3 when combined with depressed expression of AMPAR and NMDAR subunits in the *SRS* phase compared to *LP*, plus the much later timecourse of receptor changes to temporal lobe structures, strongly supports the notion that epileptogenesis continues beyond the first seizure (Sloviter, 2005; Sloviter and Bumanglag, 2013). These data indicate the importance of decreases in GABAergic inhibition in the transition to spontaneous recurrent seizures and, interestingly, very recent evidence suggests that selective loss of interneurons alone could be sufficient to elicit seizures (Chun et al., 2019).

Overall, our data, in combination with previous studies, suggest that common changes occur in epilepsy patients and other rat models of epilepsy, providing further evidence to suggest that the RISE model is a convenient and representative experimental system.

In summary, our data demonstrate that there is a consistent and reproducible change in the profile of key receptors in the RISE model of temporal lobe epileptogenesis. Changes are specific to particular receptor subunits and do not correlate with decreases in synaptic markers, suggesting that these alterations are not attributable to synaptic loss. Taken together, our results are consistent with the hypothesis that the loss of inhibitory synaptic function, perhaps in addition to, or as a consequence of, altered excitatory drive, contributes to the pathologically increased network activity in epilepsy. These results provide a platform for future investigations targeting particular receptor subunits to reduce or prevent seizures.

## Disclosures

GW and BSH received financial support from GW Pharma. The other authors declare no competing interests. We confirm that we have read the Journal's position on issues involved in ethical publication and affirm that this report is consistent with those guidelines.
