# Supplementary material for: Changes in excitatory and inhibitory receptor expression and network activity during induction and establishment of epilepsy in the rat Reduced Intensity Status Epilepticus (RISE) model
Source: Neuropharmacology. 2019 Nov 1;158:107728. doi: 10.1016/j.neuropharm.2019.107728 (PMC6892273; doi:10.1016/j.neuropharm.2019.107728)
Supplement: Multimedia component 1 [file mmc1.docx]

Supplementary Material

Changes in Excitatory and Inhibitory Receptor Expression and Network Activity During Epilepsy Induction and Establishment in the Rat Reduced Intensity Status Epilepticus (RISE) Model

Hope I. Needs*, Benjamin S. Henley*^#^, Damiana Cavallo*, Sonam Gurung,

Tamara Modebadze, Gavin Woodhall^#^ and Jeremy M. Henley

Detailed Methods

Generation of the *In Vivo* RISE Model of Epilepsy

RISE rats were generated at Aston University as reported previously {Modebadze, 2016 #21744} in strict accordance to best animal welfare practices and with approval from all local bioethics committees and the Home Office. Briefly, on day 1, rats of either sex were treated with lithium chloride (LiCl, 127 mg/kg) via subcutaneous (SC) injection followed 24 hours later by α-methyl scopolamine 1 mg/kg (SC) to reduce peripheral muscarinic cholinergic receptor activation. After 30 minutes, pilocarpine (25 mg/kg, SC) was administered to induce acute *SE* and animals monitored for seizure activity. Once a seizure scored >3 on Racine’s scale {Racine, 1972 #40372}, xylazine (2.5 mg/kg intramuscularly - IM) was immediately administered to reduce seizure severity. Rats remained in xylazine-modified SE for no more than 1 hour before the seizure activity was blocked with a cocktail (SC) containing MK-801 (0.1 mg/kg; R&D systems, UK), diazepam (2.5 mg/kg; ethanolic solution; Bayer, Germany) and MPEP (20 mg/kg; Abcam Biochemicals, UK). Rats recovered within 4-12 hours with two hourly rehydration with saline solution (5% glucose, SC). After induction of epilepsy, animals were sacrificed at relevant timepoints, with hippocampal and temporal lobe samples collected and immediately flash frozen in liquid nitrogen. Three timepoints during epilepsy progression were used:

1. *Status Epilepticus* (***SE***); rats were sacrificed 24 hours post-induction with pilocarpine.
2. Latent period (***LP***); rats were sacrificed 2-4 weeks after SE induction, corresponding to the latent, seizure-free phase of epilepsy.
3. Spontaneous recurrent seizures (***SRS***); rats were sacrificed 3+ months post-induction of SE immediately following the development of spontaneous recurrent seizures as determined by behavioural tests and/or video recording of behaviour {Modebadze, 2016 #21744}.

Corresponding samples were collected from age matched control (AMC) rats, usually litter mates, that had received no pharmacological treatments. At similar timepoints, age-matched and RISE animals were also taken for preparation of brain slices and recording of neuronal activity in brain slices *in vitro*.

Sample Preparation

We used samples prepared from the Temporal Lobe (TL) and the Hippocampus (HP). Tissue samples were crushed to a fine powder with a pestle and mortar in liquid nitrogen, resuspended directly in lysis buffer (50 mM Tris pH 7.4, 150 mM NaCl, 1% Triton, 1X Protease Inhibitor), then sonicated, vortexed briefly, and incubated on ice for 30 minutes. Samples were centrifuged, and the pellet discarded. Total protein concentration was quantified using a bicinchoninic acid (BCA) assay (Pierce ThermoFisher Scientific).

Western Blotting

Samples of hippocampus and temporal lobe dissected from four or five individual RISE and AMC rats were crushed to a fine powder with a pestle and mortar in liquid nitrogen, resuspended directly in lysis buffer (50 mM Tris pH 7.4, 150 mM NaCl, 1% Triton, 1X Protease Inhibitor), then sonicated, vortexed briefly, and incubated on ice for 30 minutes. Samples were centrifuged, and the pellet discarded. Total protein concentration was quantified using a bicinchoninic acid (BCA) assay (Pierce ThermoFisher Scientific). Equal amounts of total protein (40µg) were heated to 95°C for 10 minutes in sample buffer (2X). Samples were run in parallel on the same gel and immunoblotted along with loading controls of either β-actin, β-tubulin III or α-GAPDH, depending on the MW of the protein investigated. At least two technical repeats of SDS-PAGE and western blots were performed using the same samples. 40 μg of total protein was loaded on 10% SDS-PAGE gels, separated (150 V, 1.5 hours), and transferred onto PVDF membranes (activated with methanol) with transfer buffer (29 mM glycine, 58 mM Tris-HCl pH 7.5, 0.0375% (w/v) SDS, 20% (v/v) methanol) using a wet transfer system (400 mAmps, 70 minutes). Membranes were blocked for 1 hour in milk (5% w/v in T-PBS (10X PBS, 1% (v/v) Tween)) and incubated overnight in milk (5% (w/v) in T-PBS) containing the appropriate primary antibody (Supplemental Table 1) at 4°C. Membranes were washed extensively with T-PBS and probed with appropriate secondary HRP-antibodies (RT, 1 hour). Blots were washed extensively with T-PBS and developed using an Odyssey Fc Imaging System (LI-COR).

For reprobing, membranes were stripped by incubating for 15 minutes at 55°C with Restore PLUS Western Blot Stripping Buffer (Thermo Scientific), then washed extensively with T-PBS and blocked in milk (5% w/v in T-PBS) for 30 minutes at RT. Membranes were then incubated with the appropriate primary and secondary antibodies and developed as described above. Blots were analysed and bands quantified using Image Studio Lite (LI-COR).

Electrophysiology

Animals were taken at specific timepoints during epileptogenesis and brain slices prepared using standard techniques. A Flaming-Brown micropipette puller (Sutter Instruments, CA) was used to pull borosilicate glass microelectrodes with open tip resistance of 2-3 MΩ which were placed into area CA3 of hippocampus by viewing under a LEICA MZ6 field microscope. Activity was recorded using an EXT-01 head-stage (NPI electronics GMBH, Germany) mounted on to a manually operated micromanipulator (MM-33; Narishige, Japan) The signal was amplified 10 times using an LHBF-48X preamplifier/signal conditioner (NPI, Germany) set to band-pass filter between 700 Hz and 0.1 Hz and then amplified a further 100 times using EXT-102F amplifier (NPI, Germany). Humbug (Quest Scientific, Canada) signal conditioners were used to eliminate low amplitude line noise. The signal was simultaneously digitised using a CED 1401 digital converter onto a disk using Spike2 software.

Electrodes placed into the centre of CA3 showed spontaneous gamma oscillations (SγO), which were usually of low amplitude and spectral power. After establishment of a stable baseline SγO, slices were challenged with 100 nM kainic acid (KA), which is a near-maximal concentration for generation of stable gamma activity in CA3 in our hands. KA-induced gamma oscillations (KγO) were allowed to stabilise for 1-3 hours until regular measurements of peak power showed less than 10% change over a 20 minute period. In order to define excitability, the mean change in power was calculated by subtracting SγO power from KγO power to derive a change in power (ΔP) that could be compared across recordings. In most cases, the ΔP was on the order of 3-4 orders of magnitude, reflecting the sensitivity of CA3 to KA and providing a robust measure of glutamate receptor mediated ‘excitability’ of CA3.

**Statistical Methods**

GraphPad Prism 7 software was used to perform statistical analysis on results of all quantitative experiments, as detailed in figure legends. One sample t-tests were used to calculate and determine statistical significance between observed differences. Error bars display the standard deviation (SD). A p-value of less than 0.05 was considered significant for all experiments (p<0.05 = *, p<0.01 = **, p<0.001 = ***, p<0.0001 = ****). Multiple linear regression analysis was carried out for significantly altered proteins compared to pre- or post- synaptic marker proteins to analyse correlation between alterations in expression levels of receptor subunits and synapse expression. For electrophysiology, comparisons between drug conditions and between control and epileptic conditions were made using Mann-Whitney non-parametric statistical analyses. Neuronal oscillations were processed in Matlab (Rev 17a, Mathworks, USA) to produce fast Fourier transforms (FFTs). The Mortlet-wavelet spectrograms are labelled with wavelet scale, which is inversely related to frequency and a commonly used output from Matlab. In addition, labels for the 20 and 40 Hz pseudo-frequencies are included to approximate where the gamma sits on the plots. The specific details are presented in the results.

| **Protein** | **MW (kDa)** | **Protein Function** | **Antibody Used** | **Host Species** | **Dilution** |
| --- | --- | --- | --- | --- | --- |
| **GluA1** | 100 | AMPAR subunits | EMD Millipore  AB 1504 | Rabbit Polyclonal | 1:1000 |
| **GluA2** | 100 |  | BD Pharmingen  556341 | Mouse Monoclonal | 1:1000 |
| **GluA3** | 100 |  | ALOMONE LABS  AGC-010 | Rabbit  Polyclonal | 1:1000 |
| **GluK2** | 120 | KAR subunits | EMD Millipore  04-921 | Rabbit Monoclonal | 1:1000 |
| **GluK5** | 130 |  | EMD Millipore  06-315 | Rabbit Polyclonal | 1:1000 |
| **GluN1** | 120 | NMDAR subunits | Abcam  AB 109182 | Rabbit Monoclonal | 1:1000 |
| **GluN2A** | 170 |  | EMD Millipore  AB 1555P | Rabbit Polyclonal | 1:1000 |
| **GluN2B** | 170 |  | EMD Millipore  AB 1557P | Rabbit Polyclonal | 1:1000 |
| **mGluR1α** | 125 | mGluR subtypes | EMD Millipore  AB 1551 | Rabbit Polyclonal | 1:500 |
| **mGluR5** | 130 |  | UPSTATE Biotechnology  06-451 | Rabbit Polyclonal | 1:5000 |
| **CB1** | 60 | Cannabinoid receptor 1 | University of Indiana  FP1A | Rabbit Polyclonal | 1:500 |
| **GABA_A_ β3** | 57 | GABA_A_R subunit | UC Davis 75-149 | Mouse  Monoclonal | 1:1000 |
| **PSD95** | 95 | Postsynaptic scaffold protein | EMD Millipore  AB 1596 | Mouse Monoclonal | 1:1000 |
| **Synaptophysin** | 35 | Presynaptic vesicle release | Merk  573822 | Mouse  Monoclonal | 1:1000 |
| **Gephyrin** | 80 | Postsynaptic neuronal assembly | Synaptic Systems  147111 | Mouse Monoclonal | 1:1000 |
| **β-actin** | 42 | Loading controls | Sigma-Aldrich  A2228 | Mouse Monoclonal | 1:2000 |
| **β-tubulin III** | 55 |  | Sigma-Aldrich  090 M4775 | Rabbit  Polyclonal | 1:2000 |
| **α-GAPDH** | 35 |  | ABCAM  AB 9484 | Mouse Monoclonal | 1:20000 |

**Supplemental Table 1: Protein targets, suppliers, catalogue numbers and dilutions of the antibodies used.**

| **Hippocampus** | | | | | | |
| --- | --- | --- | --- | --- | --- | --- |
| **Independent Variable** | **Dependent Variable** | **P-value** | **Significant?** | **95% CI**  **Lower** | **95% CI**  **Upper** | **R^2^** |
| PSD95 | GluA1 | 0.800 | No | -2.054 | 1.952 | 0.0955 |
|  | GluA2 | 0.5033 | No | -2.711 | 2.319 | 0.4948 |
|  | GluA3 | 0.6387 | No | -1.319 | 1.193 | 0.2890 |
|  | GluK2 | 0.2017 | No | -0.553 | 0.9029 | 0.9029 |
|  | GluN2A | 0.4591 | No | -1.409 | 1.178 | 0.5641 |
|  | mGluR1⍺ | 0.4735 | No | -1.964 | 2.331 | 0.5416 |
| Synaptophysin | GluK2 | 0.2547 | No | -11.43 | 7.845 | 0.8483 |
| Gephyrin | GABA_A_β3 | 0.1007 | No | -1.593 | 4.696 | 0.9752 |

| **Temporal lobe** | | | | | | |
| --- | --- | --- | --- | --- | --- | --- |
| **Independent Variable** | **Dependent Variable** | **P-value** | **Significant?** | **95% CI**  **Lower** | **95% CI**  **Upper** | **R^2^** |
| PSD95 | GluA1 | 0.4164 | No | -15.64 | 19.22 | 0.6299 |
|  | GluA2 | 0.9073 | No | -11.44 | 11.18 | 0.0211 |
|  | GluA3 | 0.8036 | No | -3.651 | 3.839 | 0.0922 |
|  | GluK5 | 0.9406 | No | -22.38 | 22.72 | 0.0087 |
|  | GluN1 | 0.4777 | No | -21.28 | 25.21 | 0.5351 |
|  | GluN2A | 0.4993 | No | -15.23 | 13.00 | 0.5011 |
|  | GluN2B | 0.1198 | No | -0.920 | 2.216 | 0.9650 |
|  | mGluR1⍺ | 0.2189 | No | -1.621 | 2.534 | 0.8864 |
|  | mGluR5 | 0.4047 | No | -10.28 | 12.74 | 0.6474 |
| Synaptophysin | GluK5 | 0.6595 | No | -30.31 | 33.27 | 0.2598 |
| Gephyrin | GABA_A_β3 | 0.6810 | No | -1.219 | 1.118 | 0.2307 |

**Supplemental Table 2: Multiple linear regression analysis using PSD95, synaptophysin, and gephyrin as independent variables in analysis of significantly altered proteins.** Multiple linear regression analysis was carried out for significantly altered proteins compared to pre- or post- synaptic marker proteins to analyse correlation between alterations in expression levels of receptor subunits and synapse expression. CI = Confidence Interval.


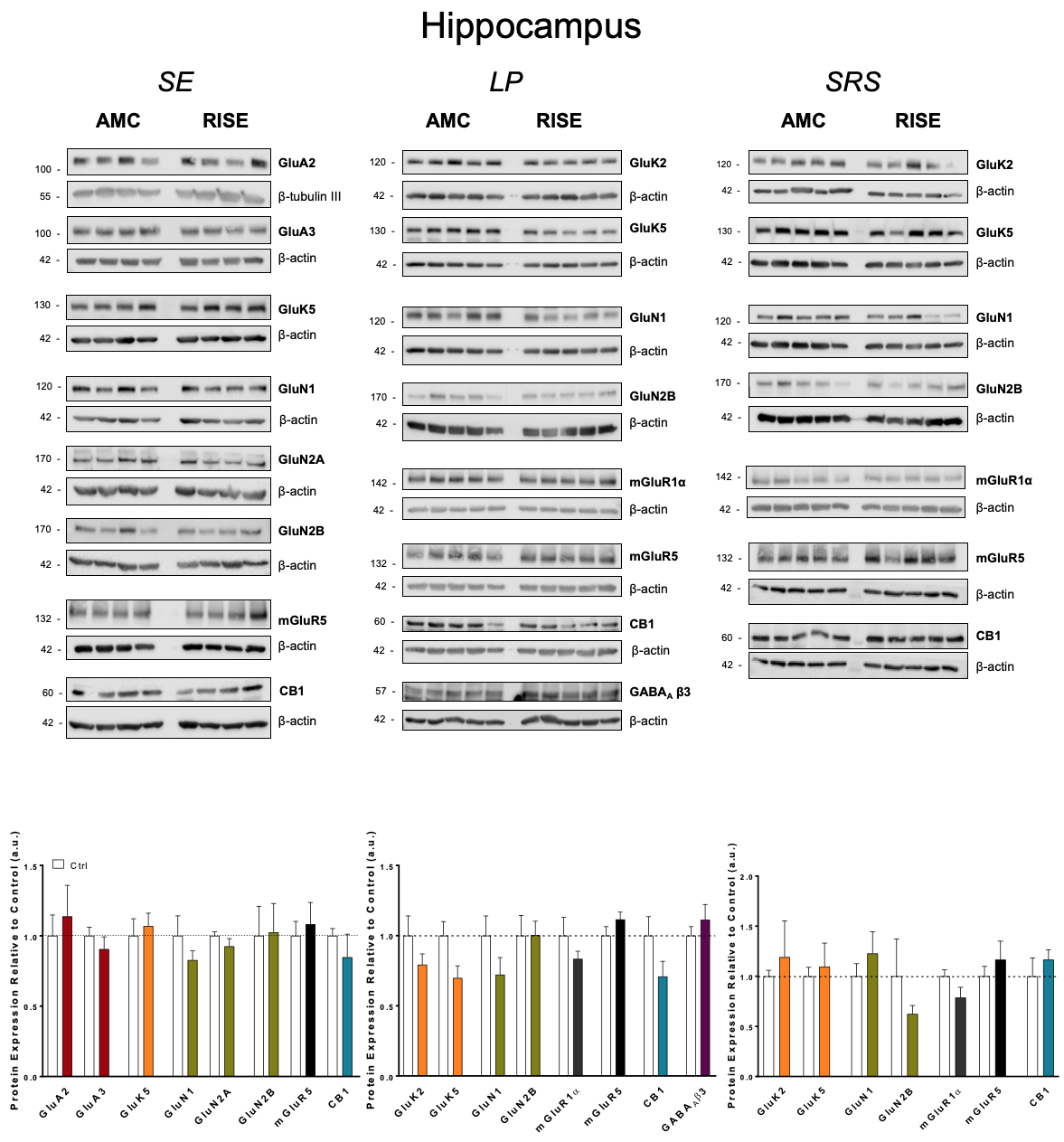


**Supplemental Figure 1: Levels of receptor proteins in hippocampus that did not showed significant changes between AMC and RISE rats at different stages of epileptogenesis.**

Top panels: Representative western blots of hippocampal samples immunoblotted with antibodies indicated. β-tubulin III, β-actin and α-GAPDH were used as loading controls. Bottom panels: Quantification of immunoreactive bands normalised to their respective loading controls. Bars represent the mean ± SD from 4-5 samples, with the mean of the control group set to 1.


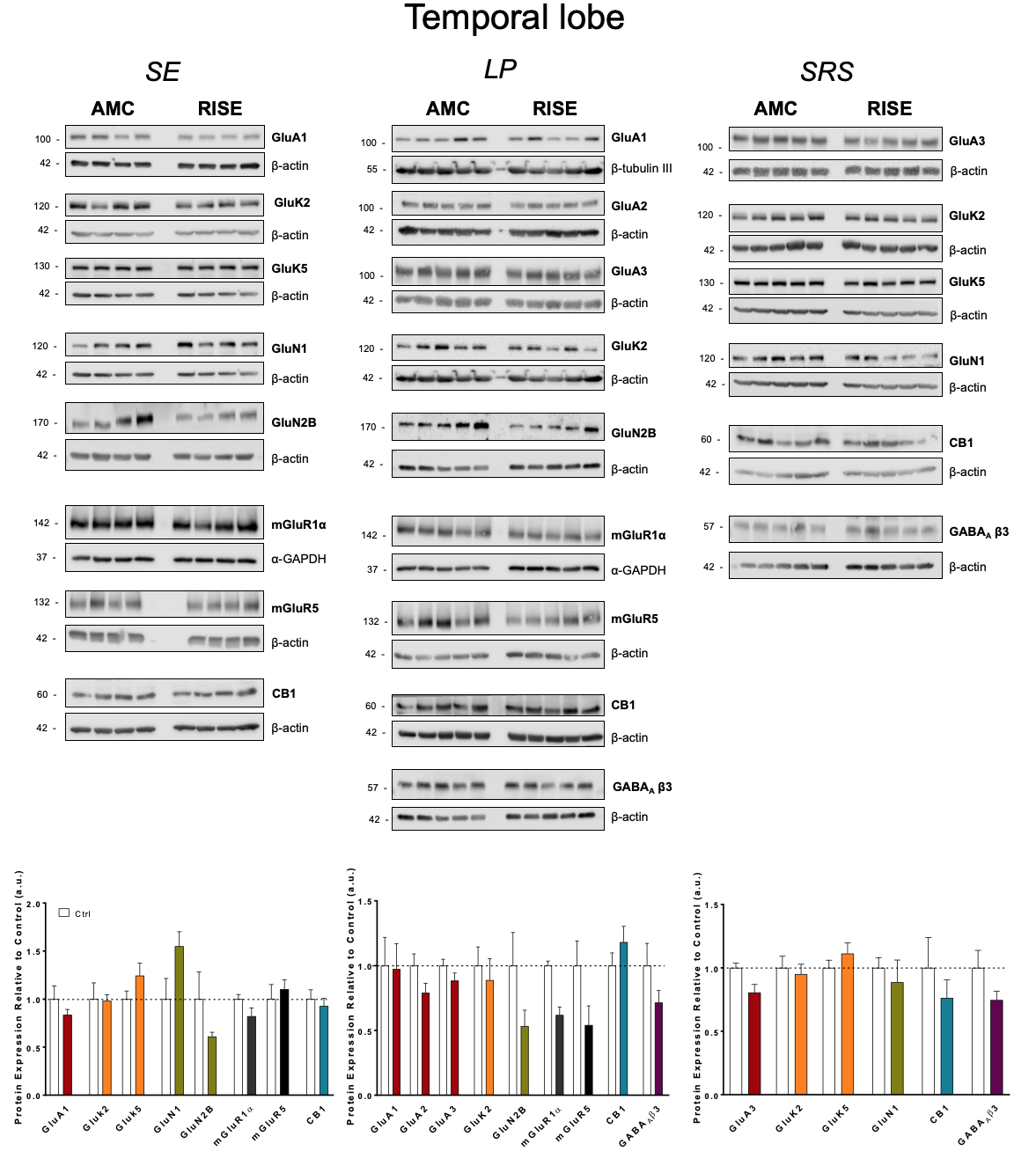


**Supplemental Figure 2: Levels of receptor proteins in temporal lobe that did not showed significant changes between AMC and RISE rats at different stages of epileptogenesis.**

Top panels: Representative western blots of hippocampal samples immunoblotted with antibodies indicated. β-tubulin III, β-actin and α-GAPDH were used as loading controls. Bottom panels: Quantification of immunoreactive bands normalised to their respective loading controls. Bars represent the mean ± SD from 4-5 samples, with the mean of the control group set to 1.

**Supplemental Figure 3: Full, untruncated representative images of hippocampal blots at different stages of epilepsy using the indicated antibodies.** Note, blots were ponceau stained and cut at indicated positions (blue arrow) to allow analysis of proteins of different Mr values and loading controls from the same blot.

**Supplemental Figure 4: Full, untruncated representative images of temporal lobe blots at different stages of epilepsy using the indicated antibodies.** Note, blots were ponceau stained and cut at indicated positions (blue arrow) to allow analysis of proteins of different Mr values from the same blot.
